# An Overview of the Statistical Methods Used for Inferring Gene Regulatory Networks and Protein-Protein Interaction Networks

**DOI:** 10.1155/2013/953814

**Published:** 2013-02-21

**Authors:** Amina Noor, Erchin Serpedin, Mohamed Nounou, Hazem Nounou, Nady Mohamed, Lotfi Chouchane

**Affiliations:** ^1^Electrical and Computer Engineering Department, Texas A&M University, College Station, TX 77843-3128, USA; ^2^Chemical Engineering Department, Texas A&M University at Qatar, 253 Texas A&M Engineering Building, Education City, P.O. Box 23874, Doha, Qatar; ^3^Electrical Engineering Department, Texas A&M University at Qatar, 253 Texas A&M Engineering Building, Education City, P.O. Box 23874, Doha, Qatar; ^4^Department of Genetic Medicine, Weill Cornell Medical College in Qatar, P.O. Box 24144, Doha, Qatar

## Abstract

The large influx of data from high-throughput genomic and proteomic technologies has encouraged the researchers to seek approaches for understanding the structure of gene regulatory networks and proteomic networks. This work reviews some of the most important statistical methods used for modeling of gene regulatory networks (GRNs) and protein-protein interaction (PPI) networks. The paper focuses on the recent advances in the statistical graphical modeling techniques, state-space representation models, and information theoretic methods that were proposed for inferring the topology of GRNs. It appears that the problem of inferring the structure of PPI networks is quite different from that of GRNs. Clustering and probabilistic graphical modeling techniques are of prime importance in the statistical inference of PPI networks, and some of the recent approaches using these techniques are also reviewed in this paper. Performance evaluation criteria for the approaches used for modeling GRNs and PPI networks are also discussed.

## 1. Introduction

Postgenomic era is marked by the availability of a deluge of genomic data and has, thus, enabled the researchers to look towards new dimensions for understanding the complex biological processes governing the life of a living organism [1–5]. The various life sustaining functions are performed via a collaborative effort involving DNA, RNA, and proteins. Genes and proteins interact with themselves and each other and orchestrate the successful completion of a multitude of important tasks. Understanding how they work together to form a cellular network in a living organism is extremely important in the field of molecular biology. Two important problems in this considerably nascent field of computational biology are the inference of gene regulatory networks and the inference of protein-protein interaction networks. This paper first looks at how the genes and proteins interact with themselves and then discusses the inference of an integrative cellular network of genes and proteins combined.

**Figure 1:**
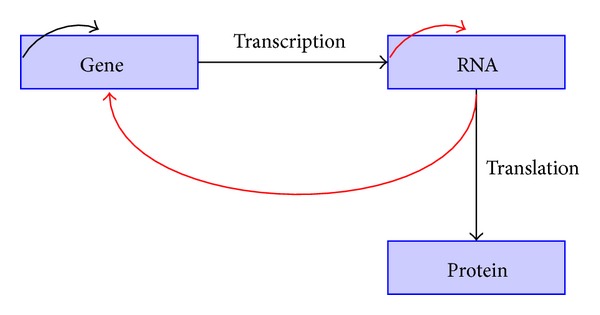
Central dogma of molecular biology.

Gene regulation is one of the many fascinating processes taking place in a living organism whereby the expression and repression of genes are controlled in a systematic manner. With the help of the enzyme RNA polymerase, DNA transcribes into mRNA which may or may not translate into proteins. It is found that in certain special cases mRNA is reverse-transcribed to DNA. The processes of transcription and translation are schematically represented in Figure 1, where the interactions in black show the most general framework and the interactions depicted in red occur less frequently. Transcription factors (TFs), which are a class of proteins, play the significant role of binding onto the DNA and thereby regulate their transcription. Since the genes may be coding for TFs and/or other proteins, a complex network of genes and proteins is formed. The level of activity of a gene is measured in terms of the amount of resulting functional product, and is referred to as gene expression. The recent high-throughput genomic technologies are able to measure the gene expression values and have provided large-scale data sets, which can be used to obtain insights into how the gene networks are organized and operated. One of the most encountered representations of gene regulatory networks is in terms of a graph, where the genes are depicted by its nodes and the edges represent the interactions between them.

The gene regulatory network (GRN) inference problem consists in understanding the underlying system model [6–10]. Simply stated, given the gene expression data, the activation or repression actions by a set of genes on the other genes need to be identified. There are several issues associated with this problem, including the choice of models that capture the gene interactions sufficiently well, followed by robust and reliable inference algorithms that can be used to derive decisive conclusions about the network. The inferred networks vary in their sophistication depending on the extent and accuracy of the prior knowledge available and the type of models used in the process. It is also important that the gene networks thus inferred should possess the highly desirable quality of reproducibility in order to have a high degree of confidence in them. A sufficiently accurate picture of gene interactions could pave the way for significant breakthroughs in finding cures for various genetic diseases including cancer.

Protein-protein interactions (PPIs) are of enormous significance for the workings of a cell. Insights into the molecular mechanism can be obtained by finding the protein interactions with a high degree of accuracy [11, 12]. The protein interaction networks not only consist of the binary interactions, rather, in order to carry out various tasks, proteins work together with cohorts to form protein complexes. It should be emphasized that a particular protein may be a part of different protein complexes, and hence the inference problem is much more complicated. The existing high-throughput proteomic data sets enable the inference of protein-protein interactions. However, it is found that the protein-protein interactions obtained by using different methods may not be equivalent, indicating that a large number of false positives and negatives are present in the data. Similar to the representation of gene regulatory networks, protein-protein interaction networks will also be modeled in terms of graphs, where the proteins denote the nodes and the edges signify whether an interaction is present between the adjacent nodes.

Many statistical methods have been applied extensively to solve various bioinformatics problems in the last decade. There are several papers that provide excellent review of various statistical and computational techniques for inferring genomic and proteomic networks [2, 12]. However, it is important to understand the fundamental similarities and differences that characterize the two inference problems. This paper provides an overview of the most recent statistical methods proposed for the inference of GRNs and PPI networks. For gene network inference, three large classes of modeling and inferencing techniques will be presented, namely, probabilistic graphical modeling approaches, information theoretic methods, and state-space representation models. Clustering and probabilistic graphical modeling methods which comprise the largest class of statistical methods using PPI data are reviewed for the protein-protein interaction networks. Through a concise review of these contemporary algorithms, our goal is to provide the reader with a sufficiently rich understanding of the current state-of-the-art techniques used in the field of genomic and proteomic network inference.

The rest of this paper is organized as follows.  Section 2 describes some of the data sets available for the inference of genomic and proteomic networks.  Section 3 reviews the recent statistical methods employed to infer gene regulatory networks. Protein-protein network inferencing techniques are reviewed in Section 4. The methods for obtaining an integrated network with gene network and protein-protein as subnetworks are given in Section 5. The inferred network evaluation is discussed in Section 6. Finally, conclusions are drawn in Section 7.

## 2. Available Biological Data

The postgenomic era is distinguished by the availability of huge amount of biological data sets which are quite heterogenous in nature and difficult to analyze [3]. It is expected that these data sets can aid in obtaining useful knowledge about the underlying interactions in gene-gene and protein-protein networks. This section reviews some of the main types of data used for the inference of genomic and proteomic networks, including, gene expression data, protein-protein interaction data, and ChIP-chip data.

### 2.1. Gene Expression Data

Of all the available datasets, gene expression data is the most widely used for gene regulatory network inference. Gene expression is the process that results in functional transcripts, for example, RNA or proteins, while utilizing the information coded on the genes. The level of gene expression is an important indicator of how active a gene is and is measured in the form of gene expression data. Similarity in the gene expression profiles of two genes advocates some level of correlation between them. In this paper, the gene expression data is denoted by means of a random variable **x**(*t*), where *t* stands for the time index.

#### 2.1.1. cDNA-Microarray Data

One way of generating cDNA-microarray data is via the DNA microarray technology, which is by far the most popular method employed for this purpose. The number of data samples is in general much smaller than the number of genes. A main drawback associated with cDNA-microarray data is the noise in the observed gene expressions. Although the gene expression values should be continuous, the inability to measure them accurately suggests the use of discretized values.

#### 2.1.2. RNA-Seq Data

**Figure 2:**
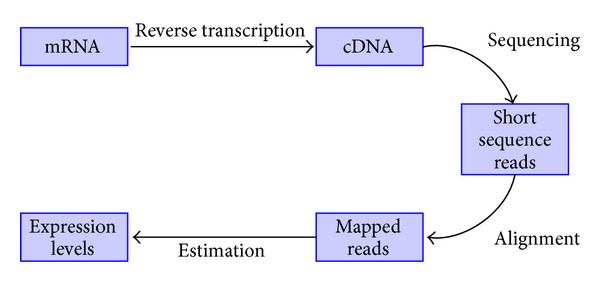
Expression estimation in RNA-Seq.

The recent advancement of sequencing technologies has provided the ability to acquire more accurate gene expression levels [13]. RNA-Seq is a novel technology for mapping and quantifying transcriptomes, and it is expected to replace all the contemporary methods because of its superiority in terms of time, complexity, and accuracy. The gene expression estimation in RNA-Seq begins with the reverse transcription of RNA sample into cDNA samples, which undergo high-throughput sequencing, resulting in short sequence reads. These reads are then mapped to the reference genome using a variety of available alignment tools. The gene expression levels are estimated using the mapped reads, and several algorithms have been proposed in the recent literature to find efficient and more accurate estimates of the gene expression levels. This process is summarized in Figure 2. The gene expression data obtained in this manner has been found to be much more reproducible and less noisy as compared to the cDNA microarrays. The next subsection describes the data used for PPI network inference.

### 2.2. Protein-Protein Interaction Data

Large-scale PPI data have been produced in recent years by high-throughput technologies like yeast two-hybrid and tandem affinity purification, which provide stable and transient interactions, and mass spectrometry, which indicates the protein complexes [11, 12]. These data sets, in addition to being incomplete also consist of false positives, and, therefore, the interactions found in various data sets may not agree with each other. Owing to this disagreement, it is imperative to make use of statistical methods to infer the PPI networks by finding reliable and reproducible interactions and predict the interactions not found yet in the currently available data.

### 2.3. ChIP-Chip Data

ChIP-chip data, which is an abbreviation of chromatin immunoprecipitation and microarray (chip), investigates the interactions between DNA and proteins. This data provides information about the DNA-binding proteins. Since some of the genes encode for transcription factors (TFs) which in turn regulate some other genes and/or proteins, this information comes in hand for the inference of gene networks [10] and the integrated network. However, generating the ChIP-chip data for large genome would be technically and financially difficult.

### 2.4. Other Data Sets

Apart from the data sets described above, gene deletion and perturbation data are worth mentioning here. Perturbation data set is generated by performing an initial perturbation and then letting the system to react to it [14]. The gene expression values at the following time instants and at steady-state are measured, thereby obtaining the response of the genes to the specific perturbation which could be the increase or decrease of the expression level of all or certain genes. Gene deletion dataset, as the name indicates, involves deleting a gene and measuring the resulting expression level of other genes. This data may effectively uncover simple direct relationships [14].

## 3. Modeling and Inferring Gene Regulatory Networks

Gene regulatory networks capture the interactions present among the genes. Accurate and reliable estimation of gene networks is significantly crucial and can reap far-reaching benefits in the field of medicinal biology, for example, in terms of developing personalized medicines. The following subsections review the main statistical methods used for inference of gene regulatory networks. First, the important class of probabilistic graphical models is presented.

### 3.1. Probabilistic Graphical Modeling Techniques

Probabilistic graphical models have emerged as a useful tool for reverse engineering gene regulatory networks. A gene network is represented by a graph **G** = (*V*, *E*), where *V* represents the set of vertices (genes), and *E* denotes the set of edges connecting the vertices. The vertices of the graph are modeled as random variables and the edges signify the interaction between them. The expression value of gene *i* is denoted by *X*_*i*_, and the total number of genes in the network is denoted by *N*. The following subsections briefly describe some of the robust and popular graphical modeling techniques for gene network inference.

#### 3.1.1. Bayesian Networks

Bayesian networks model the gene regulatory networks as directed acyclic graphs (DAGs). To simplify the inference process, the probability distribution of DAG-networks is generally factored in terms of the conditional distributions of each random variable given its parents:

(1) $$\begin{array}{c} P\left(X\right)=\prod_{i=1}^{N}P\left(X_{i}\;|\;Pa\left(X_{i}\right)\right), \end{array}$$

where *Pa*(*X*_*i*_) denotes the parent of node *X*_*i*_. The gene regulatory network is inferred by using the Bayesian network learning techniques. This is done by maximizing the probability *P*(**G** | **D**), where **D** denotes the available gene expression data. Several scoring metrics have been proposed to obtain the best graph structure [15]. The network, thus, obtained is unique to the extent of equivalence class; that is, the independence relationships are uniquely identified.

The gene expression data available to date consist of very few data points, while the number of genes is substantially larger, rendering the system to be underdetermined. As an alternative to finding the complete networks, scientists have proposed looking at certain important features, for example, Markov relations and order relations. If a gene *X* is present in the minimal network blanketing the gene *Y*, then a Markov relation is said to be established. A relationship between two genes is referred to as an ordered relation if a particular gene *X* appears to be a parent of another gene *Y* in all the equivalent networks. By aggregating this information, it is possible to infer the underlying regulatory structure robustly and reliably. The network structure inferred in this manner looks at the static interactions only. In order to cater for the dynamic interactions inherent in gene networks, dynamic Bayesian networks (DBNs) have been used [16, 17].

#### 3.1.2. Qualitative Probabilistic Networks

**Figure 3:**
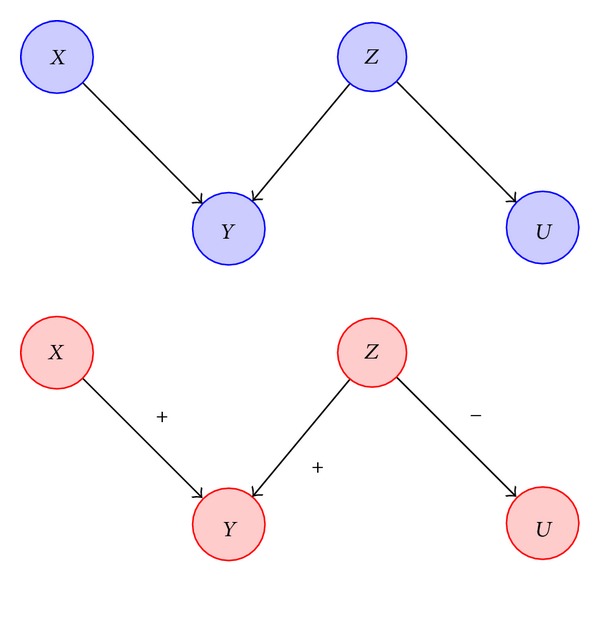
Qualitative probabilistic network (red) for a Bayesian network (blue).

A novel method of modeling gene networks is via the usage of qualitative probabilistic networks (QPNs), which represent the qualitative analog of the DBNs [18]. The structural and independence properties of QPNs are the same as those of Bayesian networks. However, instead of being concerned about the local conditional probabilities of the random variables, the former class of models looks at how the changes in probabilities of the random variables affect the probabilities of their immediate parents. This change is measured in qualitative terms instead of quantitative values, that is, whether the probabilities increase, decrease, or stay the same as shown in Figure 3.

Two important properties of QPNs are the qualitative influences and the qualitative synergies. A positive influence denoted by *I* ^+^(*X*, *Y*) indicates the greater possibility of *Y* having a higher value when that of *X* is high and vice versa, irrespective of all other variables; that is,

(2) $$\begin{array}{c} I^{+}\left(X,Y\right)\;\text{iff}\;\;P\left(y\;|\;x,W\right)>P\left(y\;|\;-x\right). \end{array}$$

In the case of three variables, QPNs look at the synergies. A positive additive synergy, denoted by *S* ^+^({*X*, *Y*}, *Z*), exists when the combined effect of the parent nodes is greater on the child node than their individual effects given by

(3) S+({X,Y},Z) iff  P(z ∣ x,y,W)+P(z ∣ −x,−y,W)>P(z ∣ x,−y,W)+P(z ∣ −x,y,W).

QPNs, thus, provide more insight into the gene networks by indicating whether a particular gene is a promoter or an inhibitor.

#### 3.1.3. Graphical Gaussian Models

Graphical Gaussian models, also known as covariance selection or concentration graph models, provide a simple and effective way of characterizing the gene interactions [19, 20]. This method relies on assessing the conditional dependencies among genes in terms of partial correlation coefficients among the gene expressions and results in an undirected network. A covariance matrix is estimated using the available gene expression data sets. Suppose that **X** ∈ ℝ^*n*×*n*^ denotes the gene expression data matrix, where the rows correspond to observations and the columns correspond to genes, then an estimate of the covariance matrix is obtained by

(4) $$\begin{array}{c} \hat{W}=\frac{1}{N-1}X^{T}X. \end{array}$$

Assuming invertibility of $\hat{W}$, the partial correlations can be determined as

(5) $$\begin{array}{c} {\hat{\rho }}_{ij}=-\frac{{\hat{w}}_{ij}}{\sqrt{{\hat{w}}_{ii}{\hat{w}}_{jj}}}, \end{array}$$

where ${\hat{\rho }}_{ij}$ denotes the partial correlation between genes *i* and *j*.

#### 3.1.4. Graphical LASSO Algorithm

A major drawback of the covariance-matrix-estimation-based methods is their unreliability due to the small number of data samples. Making use of the fact that gene networks are inherently sparse, it is possible to obtain the dependencies between genes by means of a penalized linear regression approach [20]. The graphical Least Absolute Shrinkage and Selection Operator (LASSO) algorithm solves the network inference problem efficiently by maximizing the following penalized likelihood function:

(6) $$\begin{array}{c} \frac{2}{n}l\left(W\right)=\log\left(\det\left(W\right)\right)-\text{trace}\left(\hat{W}W\right)-\rho {||W||}_{1}, \end{array}$$

where *ρ* controls the sparsity of the network, notation ||·||_1_ represents the *l*_1_-norm, and **W** denotes the covariance matrix. This minimization can be carried out by using block gradient descent methods, the details of which can be found in [20] and the references therein.

### 3.2. State-Space Representation Models

**Figure 4:**
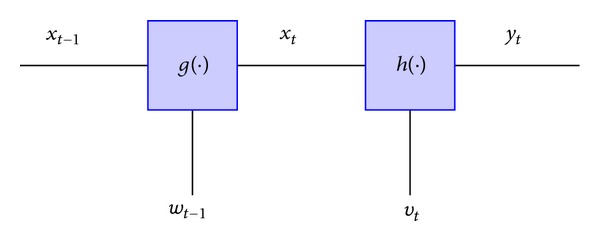
State-Space model.

One of the earliest and widely used methods of modeling gene networks is by employing the state-space representation models [21]. As opposed to other classes, all the methods belonging to this class model the dynamic evolution of the gene network. These models generally consist of two sets of equations, the first set of equations representing the evolution of the hidden state variables denoted by **z**(*t*), and the second set of equations relating the hidden state variables with the observed gene expression data, denoted by **x**(*t*) as depicted in Figure 4. The functions *g*(·) and *h*(·) describe the evolution of hidden and observed variables, respectively. Next, in this section we will describe various models for gene network inference using the state-space representation model.

#### 3.2.1. Linear State-Space Model

The simplest model for state-space equations is the linear Gaussian model given by [21, 22]:

(7) $$\begin{array}{c} z\left(t\right)=Az\left(t-1\right)+v\left(t\right),\\ x\left(t\right)=Cz\left(t\right)+\text{w}\left(t\right), \end{array}$$

where **A** is a matrix representing the regulatory relations between the genes, and *t* stands for the discrete time points. Difference equations are used in place of differential equations because discrete observations are available in the gene expression data. The noise components **v**(*t*) and **w**(*t*) represent the system and the measurement noise, respectively, and are assumed to be Gaussian. The noise models the uncertainty present in the estimated gene expression data. The matrix **C** is generally considered to be an identity matrix. Inference in gene networks modeled by the state-space representation (7) can be performed using standard Kalman filter updates. The simplicity of the state-space model avoids overfitting of the network, and therefore, it provides reliable results.

#### 3.2.2. Nonlinear Models

While it is useful to represent gene networks by simple models to ease the computational complexity, it is also imperative to incorporate nonlinear effects into the system equations, since the genes are known to interact nonlinearly [23]. A particular function that is frequently used to capture the nonlinear effects is the sigmoid squash function defined below in (9) [24]. The nonlinear state-space representation model capturing the gene interactions is described by the following system of equations:

(8) $$\begin{array}{c} z\left(t\right)=Az\left(t-1\right)+Bf\left(z\left(t-1\right),\mu \right)+I_{0}+v\left(t\right), \end{array}$$

where the *j*th entry of vector function *f*(·) is given by the sigmoid squash function:

(9) $$\begin{array}{c} f_{j}\left(z_{j},\mu_{j}\right)=\frac{1}{1+e^{-\mu_{j}z_{j}}}, \end{array}$$

where *μ* is a parameter to be identified. Matrix **A** represents the linear relationships between the genes, while matrix **B** characterizes the nonlinear interactions. The problem, thus, boils down to the estimation of the following unknowns in the system:

(10) $$\begin{array}{c} \theta =\left[A,B,\mu ,I_{0}\right], \end{array}$$

where **I**_0_ models the constant bias. One way of solving these equations is by using the extended Kalman filter (EKF) [24], which is a popular algorithm for solving nonlinear state-space equations. EKF algorithm provides the solution by approximating the nonlinear system by its first-order linear approximation. Other variants of Kalman filter algorithm like the cubature Kalman filter (CKF), unscented Kalman filter (UKF), and particle filter algorithm are also used to solve such inference problems [25].

However, for many studies, the considered nonlinear model is comprised of a large number of unknowns and in order to estimate these unknown variables with considerable accuracy, data sets consisting of a large number of samples are required. The availability of smaller data sets represents an insurmountable obstacle in the reliable estimation of a large number of unknowns. This problem can be partially avoided by simplifying the model to include only nonlinear terms, and thus reducing the number of unknown parameters to the bare minimum [25] and by approximating *μ* to be one. The system of equations corresponding to such a parsimonious scenario is then given by

(11) $$\begin{array}{c} z\left(t\right)=Bf\left(z\left(t-1\right)\right)+v\left(t\right), \end{array}$$

where *f* is the function defined previously.

#### 3.2.3. Models with Sparsity Constraints

A crucial feature for many gene networks is their inherent sparsity; that is, all genes in the network are connected to a few other genes only. Therefore, matrices **A** and **B** depicting the regulatory relations between the genes are expected to contain only very few nonzero values as compared to the size of these matrices. Therefore, one may apply shrinkage-based methods like LASSO [25, 26] for parameter estimation and parsimonious model selection. One of the ways for inferring models with sparsity constraints is to perform dual estimation, which involves estimating the states and the parameters one by one. The hidden states can be estimated using the particle filter algorithm, and once all the estimates for the hidden states are obtained, they can be stacked together to form a matrix and thus the following system of equations is obtained to perform the parameter estimation:

(12) $$\begin{array}{c} \left[\begin{array}{c} z_{n1}\\ z_{n2}\\ \vdots \\ z_{nI} \end{array}\right]=\left[\begin{array}{ccc} f\left(z_{0,1}\right) & \ldots & f\left(z_{0,N}\right)\\ f\left(z_{1,1}\right) & \ldots & \vdots \\ \vdots & \ddots & \\ f\left(z_{I-1,1}\right) & & f\left(z_{I-1,N}\right) \end{array}\right]\left[\begin{array}{c} b_{n1}\\ b_{n2}\\ \vdots \\ b_{nN} \end{array}\right]+\left[\begin{array}{c} v_{n1}\\ v_{n2}\\ \vdots \\ v_{nI} \end{array}\right], \end{array}$$

which can be expressed compactly in vector/matrix-form representation as

(13) $$\begin{array}{c} z_{n}=\Phi b_{n}+v_{n}. \end{array}$$

LASSO operates on this system of equations and produces a parameter vector **b**_*n*_ by minimizing the criterion [27]:

(14) $$\begin{array}{c} \underset{b_{n}}{\min}\frac{1}{2}{||z_{n}-\Phi b_{n}||}_{2}^{2}+\rho {||b_{n}||}_{1}. \end{array}$$

The parameter estimates obtained using LASSO-based algorithms appear to be more reliable than the estimates provided by other approaches [25].

#### 3.2.4. State-Space Models for Time-Delayed Dependencies

The state-space models discussed so far do not consider time delays whereas it has been found that time-delayed interactions are present in gene networks [28] due to the time required for the processes of transcription and translation to take place. One of the ways to model this phenomenon is by adopting the following state-space model:

(15) z(t)=Az(t−1)+Bu(t−τ)+v(t),x(t)=Cz(t)+w(t).

In this state-space model, the input is considered to be the expression profile of a regulator such as a transcription factor. Here, **A** stands for the *N* × *N* state transition matrix, while *N* × *p* matrix **B** captures the effect of *p* regulators on the system. The value of the time delay *τ* is obtained by finding the best fit over a range of possible values using Akaike's information criterion (AIC) in order to avoid overfitting the network.

### 3.3. Information Theoretic Methods

Information theoretic methods have provided some of the most robust and reliable algorithms for gene network inference and form the basis of a standard in this field [29–31]. A particular advantage associated with these methods is their ability to work with minimal assumptions about the underlying network. This is in contrast with the probabilistic graphical modeling techniques as well as the state-space models, both of which have their own set of assumptions. As highlighted previously, a Markov network provides an undirected network, while Bayesian networks are not able to incorporate cycles or feedback loops. State-space models apart from the linear Gaussian model make critical assumptions on the model structure. These drawbacks are not present in the case of information theoretic methods. The following discussion presents the main information theoretic approaches for inferring gene regulatory networks.

#### 3.3.1. Finding the Correlation between Genes

Two of the most fundamental concepts in information theory are mutual information and entropy. Mutual information between two random variables *X* and *Y* is defined as [32]

(16) I(X;Y)=∑x,y[p(x,y)log⁡p(x,y)p(x)p(y)]=H(X)+H(Y)−H(X,Y),

where *H* denotes the entropy or the uncertainty present in a random variable, and it is given by

(17) $$\begin{array}{c} H\left(X\right)=-\sum_{x}p\left(x\right)\log p\left(x\right). \end{array}$$

Mutual information measures the correlation between two random variables. In the context of gene network inference, a higher mutual information between two genes indicates a higher dependency, and therefore, a possible interaction between them. Some of the most important and robust algorithms for gene network inference make use of the mutual information for finding the interacting genes [29, 30].

#### 3.3.2. Identifying Indirect Interactions between Genes

**Figure 5:**
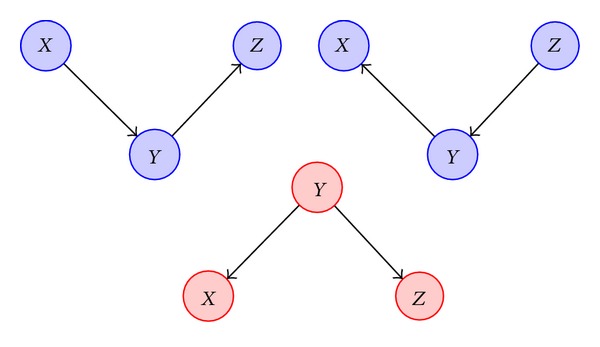
Markov chain (blue) and common cause (red).

If the mutual information between two genes is greater than a certain threshold, it indicates some correlation between them. However, this information alone is not sufficient to decide whether the genes are connected directly or indirectly via an intermediate gene. The data processing inequality (DPI) provides some insight to assess whether such a scenario holds. In case of three genes forming a Markov chain as shown in Figure 5, DPI can be expressed as

(18) $$\begin{array}{c} I\left(X;Y\right)\leq \min\left[I\left(X;Z\right),I\left(Y;Z\right)\right]. \end{array}$$

Using this inequality, it is found that the interaction with the least mutual information is an indirect one. This method is employed in ARACNE [29], which has become a standard algorithm for gene network inference. However, DPI fails to hold in situations where one of the three genes is a parent gene to the other two genes. Conditional mutual information has been proposed to be used in such cases [30]. Conditional mutual information is defined as

(19) I(X;Y ∣ Z)=∑X,Y,Z[p(x,y,z)log⁡p(x,y ∣ z)p(x ∣ z)·p(y ∣ z)]=H(X,Z)+H(Y,Z)−H(Z)−H(X,Y,Z).

If *I*(*X*; *Y* | *Z*) is much less than *I*(*X*; *Y*), it implies that *Z* is a parent of the genes *X* and *Y* as shown in Figure 5. In case the two quantities are almost equal, it means that the gene *Z* does not have any influence on the other two genes. Therefore, by employing the idea of conditional mutual information, indirect interactions in the case of common cause can be sifted.

#### 3.3.3. Finding the Directed Networks

Calculating the mutual information using static data does not provide any information about the directed relationships. On the other hand, using time series data may indicate the directionality of interactions as well [33]. Mutual information for time series data can be expressed as

(20) $$\begin{array}{c} I\left(X_{t+1};Y_{t}\right)=\sum_{x_{t+1},y_{t}}\left[p\left(x_{t+1},y_{t}\right)\log\frac{p\left(x_{t+1},y_{t}\right)}{p\left(x_{t+1}\right)p\left(y_{t}\right)}\right]. \end{array}$$

If a high value is obtained for *I*(*X*_*t*+1_; *Y*_*t*_), it signifies a directed relationship from gene *Y* to *X*. While using these methods, the determination of the significance threshold is of considerable importance and can be estimated based on the prior knowledge about the network.

The information theoretic quantities discussed so far are symmetric (or bidirectional) and do not provide any information about the directionality by themselves. Some new metrics have been proposed recently to infer asymmetric or one-directional relationships such as the *ϕ*-mixing coefficient defined as [34]:

(21) $$\begin{array}{c} \phi \left(Y\;|\;X\right)=\underset{S\subseteq A,T\subseteq B}{\max}\left|\text{Pr}\left\{Y\in T\;|\;X\in S\right\}-\text{Pr}\left\{Y\in T\right\}\right|. \end{array}$$

In other words, this coefficient provides a measure of independence or difference between two genes *X* and *Y*. DPI also holds true for the *ϕ*-mixing metric, and therefore, it can be used to identify the indirect interactions as in the case of mutual information.

#### 3.3.4. Time-Delayed Dependencies

Another way of finding directed relationships is by detecting the time-delayed dependencies by using time series data. The time instants at which the mutual information goes above or drops below the thresholds *τ*_up_ and *τ*_down_, respectively, are noted [35]. These instants are called the initial change of expression (IcE) times and are defined as

(22) $$\begin{array}{c} \text{IcE}\left(x_{a}\right)=\underset{j}{\arg\;\min}\left\{\frac{x_{a}^{j}}{x_{a}^{0}}\geq \tau_{\text{up}}\;\text{or}\;\frac{x_{a}^{j}}{x_{a}^{0}}\leq \tau_{\text{down}}\right\}. \end{array}$$

It can be seen that a gene *x*_*a*_ can be a regulator for gene *x*_*b*_ if and only if (iff) IcE(*x*_*a*_) < IcE(*x*_*b*_). The mutual information in this case is given by

(23) $$\begin{array}{c} I^{k}\left(x_{a};x_{b}\right)=\sum_{i=1}\left[p\left(x_{a}^{i},x_{b}^{i+k}\right)\log\frac{p\left(x_{a}^{i},x_{b}^{i+k}\right)}{p\left(x_{a}^{i}\right)p\left(x_{b}^{i+k}\right)}\right], \end{array}$$

where the delay is denoted by *k*. The next step consists in finding the maximum of the mutual information values calculated for all the time delays; that is,

(24) $$\begin{array}{c} I\left(x_{a},x_{b}\right)=\underset{k}{\max}\left\{I^{k}\left(x_{a},x_{b}^{\left(k\right)}\right)\right\}\\ \text{for}\;k=1,2,\ldots ,\text{while}\;\text{IcE}\left(x_{a}\right)\leq \text{IcE}\left(x_{b}\right). \end{array}$$

If the value of the maximum mutual information is greater than a prespecified threshold, it is concluded that a directed relationship exists from *x*_*a*_ to *x*_*b*_. The calculation of threshold is very important in all the information theoretic methods which is selected on the basis of the predetermined *P*-value [29]. This helps to obtain networks with the required significance value.

#### 3.3.5. Model Selection

An important and necessary step in the implementation of the above-mentioned algorithms is the model selection. A network formed by using mutual information alone will result in an overfitted structure, and therefore, model selection becomes imperative. Minimum description length (MDL) principle was proposed as a general approach for model selection. MDL states that the network with the shortest coding length should be selected. For a network with a large number of nodes, the coding length will be large and vice versa. MDL principle provides a trade-off and aids in selecting only the significant interactions between the genes. MDL was applied in various ways in finding the coding length of the network and the probability densities associated with it [33]. Another way of using this principle is in conjunction with the maximum likelihood (ML) principle which results in a more general algorithm [36]. Further details on this algorithm can be found in [36]. Thus, it appears that the tools of information theory are quite powerful in modeling and inferring gene regulatory networks.

## 4. Inferring the Protein-Protein Interaction Networks

Having examined the gene network inference problem, this section describes the statistical methods that are used to find reliable and complete protein-protein interaction networks. As opposed to gene networks which are mostly inferred using the expression data or the likes of it, inference of PPI networks can be carried out in various ways such as phylogenetic profiling and identification of structural patterns. This paper focuses only on the methods that employ PPI data to make inference. The given data in this scenario are the protein-protein interactions. However, such data sets consist of a large number of false positives and negatives and are far from being complete and homogeneous. Therefore, only a small overlap is found between the PPI data sets obtained from various sources. However, it is observed that the interactions predicted by more than one method are more reliable [37]. One of the challenges is the large number of interactions indicated by the PPI data as opposed to the considerably fewer interactions assumed to be present in reality. Therefore, the problem in this scenario is to find more reliable interactions and predict the yet unknown interactions. In addition, the protein interactions can be of different types ranging from stable ones to transient ones [37].

It is to be noted that as opposed to the gene networks, a lot of work can still be done for protein-protein network inference using the probabilistic methods. In a living organism, several proteins work together to carry out various tasks forming a protein complex. Most of the PPI data consists of binary interactions only and it is very rare to find interactions between more than two proteins simultaneously. Hence, identification of protein complexes is of prime importance to gain a better understanding of the cellular network.

Detecting protein complexes is a fundamental area of study of protein networks [38], for which various clustering methods were applied. One of the various ways of identifying the protein complexes include graph segmentation, where the graph is clustered into subgraphs using cost-based search algorithms. Another approach is broadly categorized as conservation across species [38], where alignment tools are used to find the complexes that are common in multiple data sets coming from different species. In what follows, some of the recently proposed probabilistic graphical-modeling- and clustering-based methods are described.

### 4.1. Markov Networks

The available PPI data look mostly at the binary interactions, and interactions of three or more genes are hard to find. However, it is important to look at the interacting proteins holistically. Markov networks are probabilistic graphical modeling techniques which result in undirected graphs. Suppose **X** = {*X*_1_,…, *X*_*N*_} is a vector of random variables modeling the proteins. Their joint distribution is captured in terms of the potentials *ψ*_*c*_ ∈ Ψ. The random variables **X**_*c*_ that are connected to each other are called the scope for the particular potential *ψ*_*c*_. The joint probability distribution is then given by

(25) $$\begin{array}{c} P\left(X=\text{x}\right)=\frac{1}{Z}\prod_{c\in C}e^{\psi_{c}(x_{c})}, \end{array}$$

where *Z* is the normalizing constant also called the partition function. In this way, a compact representation of the probability distribution is obtained. The network structure is learned by using the independence properties of Markov networks using the available PPI data. The details of this method can be found in [37].

### 4.2. Bayesian Networks

Another way of modeling PPI networks is by means of Bayesian networks (BNs) [39], which represent a probabilistic graphical modeling technique. The inference algorithm is based on finding the conditional probability densities *P*(*X*_*i*_ | *C*), where *C* denotes the class variable, and *X*_*i*_ denotes the *i*th node in the network. A particular strength of BNs is their ability to estimate model parameters even in the presence of incomplete data, which is often the case with the PPI networks. This fact makes BN a perfectly suited method for modeling protein networks. One way of estimating the model parameters is via the Expectation Maximization (EM) algorithm [39]. The joint probability distribution is expressed as

(26) $$\begin{array}{c} P\left(C,X_{1},\ldots ,X_{N}\right)=P\left(C\right)\prod_{i}P\left(X_{i}\;|\;C\right). \end{array}$$

Assuming all the random variables to be independent of each other, the posterior density is given by

(27) $$\begin{array}{c} P\left(C\;|\;X_{1},\ldots ,X_{N}\right)=P\left(C\right)\prod_{i}\frac{P\left(X_{i}\;|\;C\right)}{P\left(X_{i}\right)}. \end{array}$$

Once the model parameters are known, prediction can be made about random variables for which the data may not be available. Therefore, this algorithm provides a suitable method for finding protein complexes.

### 4.3. Graphical Clustering Methods

One of the ways of graph clustering is based on supervised learning [12, 38]. The subgraphs are modeled using Bayesian networks, and the features consist of topological patterns of graphs and biological properties. Rather than assuming the widely used cliqueness property, which considers all the nodes to be connected with each other, the algorithm looks for the properties that are inferred from already known complexes. Two important features are the label *C* indicating whether a subgraph is a complex and the number of nodes *N*. The other feature descriptors including degree statistics, graph density, and degree correlation statistics are indicated by *X*_1_,…, *X*_*m*_ and are considered independent given *C* and *N*. The number of nodes in and off itself is an important feature. Its importance can be seen from the fact that a larger number of nodes in a subgraph indicate a lesser probability of it being a clique. All the subgraphs are assigned scores by making use of these properties. One way of finding how probable it is for a subgraph to be a protein complex is to perform simple hypothesis testing by calculating the following conditional probability [12, 38]:

(28) L=log⁡p(c1 ∣ x1,…,xm)p(c0 ∣ x1,…,xm)=log⁡p(n ∣ c1)∏k=1mp(xk ∣ n,c1)p(n ∣ c0)∏k=1mp(xk ∣ n,c0),

where the posterior probabilities are calculated via Bayes rule as

(29) p(ci ∣ n,x1,…,xm)   =p(n,x1,…,xm ∣ ci=1)p(ci=1)p(n,x1,…,xm)   =p(x1,…,xm ∣ n,ci=1)p(n ∣ ci=1)p(ci=1)p(n,x1,…,xm).

These probability densities can be calculated using maximum likelihood methods. By comparing the obtained score to a predetermined threshold, some of the subgraphs can be labeled to be complexes. This algorithm takes the weighted matrix of PPI data as input, where the weights are assigned using the likelihood of any particular interaction. Several other graphical-clustering-based methods are surveyed in [12].

### 4.4. Matrix Factorization Methods for Clustering

Nonnegative matrix factorization (NMF) is a method widely used in problems of clustering. Application of this technique has been proposed recently in [40], where an ensemble of nonnegative factored matrices obtained using protein-protein interaction data are combined together to perform soft clustering. The importance of this step lies in the fact that a particular object may belong to multiple classes. Hence, the various algorithms reported in the literature performing hard clustering may not be of much benefit in such scenarios. This ensemble NMF method is observed to classify the proteins in accordance with the functions they perform and also identify the multiple groups they belong to.

The algorithm produces *τ* base clusterings by factorizing the symmetric data matrix *S* of protein interactions in the following manner [40]:

(30) $$\begin{array}{c} \underset{V>0}{\min}{||S-VV^{T}||}_{F}^{2}, \end{array}$$

where ||·||_*F*_ denotes the Frobenius norm. The factors **V** produced in this manner are not unique. Let *k*_*i*_ be the number of clusters in the *i*th base cluster, each with a different value in order to promote diversity. Once the ensemble of factored matrices is available, the next step is to construct the graph by combining the information present in them. Parameter *l* = *k*_1_ + ⋯+*k*_*τ*_ gives the total number of basis vectors which are denoted by **V** = {**v**_1_,…, **v**_*l*_}. Each vector denotes a node on the graph, and the edge weight is calculated using the Pearson correlation for a pair of vector (**v**_*i*_, **v**_*j*_) given by

(31) $$\begin{array}{c} \text{cor}\left(v_{i},v_{j}\right)=\frac{1}{2}\left(\frac{{\left(v_{i}-{\overset{-}{v}}_{i}\right)}^{T}\left(v_{j}-{\overset{-}{v}}_{j}\right)}{{||v_{i}-{\overset{-}{v}}_{i})||}_{2}\cdot {||v_{j}-{\overset{-}{v}}_{j}||}_{2}}+1\right). \end{array}$$

Having looked at the GRNs and PPI network inference problems individually, we now proceed to review the recent advancements in the joint modeling of the two networks.

## 5. An Integrated Cellular Network

**Figure 6:**
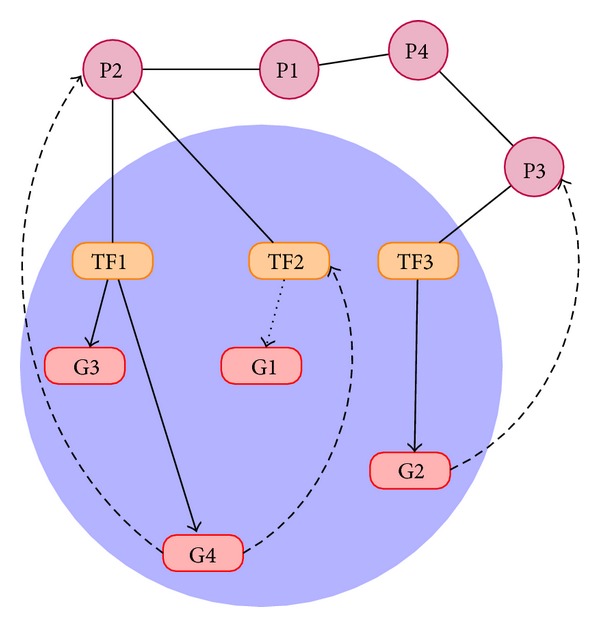
An integrated cellular network.

The advances in reverse engineering of GRNs and PPI networks have paved the way for joint estimation of GRNs and PPI networks [41]. This is a step towards the inference of an integrated network consisting of genes, proteins, and transcription factors, indicating interactions among themselves and each other.  Figure 6 shows the schematic of an integrated cellular network. In this section, we review two important ways of estimating a joint network.

### 5.1. Probabilistic Graphical Models for Joint Inference

Reference [41] proposed an interesting method for estimating GRNs and PPI networks simultaneously. Suppose that the gene expression is denoted by **x** and PPI data is represented by **y**. The algorithm provides an undirected protein network *G*_*p*_ and a directed gene network *G*_*r*_, modeled using Markov and Bayesian networks, respectively, by maximizing their joint distribution; that is,

(32) P(Gr,Gp ∣ X,Y)∝P(Gr,Gp,X,Y)=P(X ∣ Gr)P(Y ∣ Gp)P(Gr,Gp),

where *P*(*X* | *G*_*r*_, *G*_*p*_) = *P*(*X* | *G*_*r*_) and *P*(*Y* | *Gr*, *G*_*p*_) = *P*(*Y* | *G*_*p*_). The inference on Markov and Bayesian networks is performed in the same manner as explained in the previous sections. The two subnetworks are estimated iteratively till the algorithm converges. Further details on this algorithm can be found in [41].

### 5.2. Joint Estimation Using State-Space Model

State-space model can also be used to obtain an integrated network of gene and protein-protein interactions [42, 43]. A novel approach employing nonlinear model is proposed in [43], where the system parameters are estimated using constrained leastsquares. The gene expression is assumed to follow a dynamic model given by

(33) $$\begin{array}{c} x_{i}\left(t+1\right)=x_{i}\left(t\right)+\sum_{j=1}^{N}a_{ij}s_{i}\left(t\right)-\lambda_{i}z_{i}\left(t\right)+k_{i}+w_{i}\left(t\right), \end{array}$$

where

(34) $$\begin{array}{c} s_{j}\left(t\right)=f_{i}\left(y_{j}\left(t\right)\right)=\frac{1}{1+\exp\left\{-\left(y_{j}\left(t\right)-\mu_{j}\right)/\sigma_{j}\right\}}, \end{array}$$

and *y*_*j*_ denotes the protein activity profile of *j*th transcription factor, and its mean and standard deviations are represented by *μ*_*j*_ and *σ*_*j*_, respectively. The magnitude of *a*_*ij*_ indicates the strength of relationship between the *j*th TF and *i*th gene, and the sign suggests whether it is an excitatory or inhibitory relationship. The model in (33) suggests that the gene expression level at *t*th time instant depends upon the gene expression level at the previous time instant as well as the protein activity level. The degradation effect of gene expression is modeled by *λ*_*i*_, *k*_*i*_ is a constant representing the basal level, and *w*_*i*_(*t*) is the Gaussian noise modeling the uncertainties in the model and the errors in the data.

The protein activity level follows the following dynamic model:

(35) yn(t+1)=yn(t)+∑m=1Mbnmyn(t)ym(t) +αnxn(t)−βnyn(t)+hn+νn(t),

where *b*_*ij*_ gives the relationship between the proteins, *α*_*n*_ indicates the translation effect of mRNA to protein, and *ν*_*n*_(*t*) is the Gaussian noise. The unknown parameters for both the models are given by

(36) θi=[ai1⋯aiNλiki]T,  ϕn=[bn1⋯bnMαnβnhn]T

and are estimated by solving a constrained least squares problem [43]. Once the individual subnetworks are obtained, they are merged together to form one cellular network with the TFs connecting them together.

The problem of inferring an integrated network is in relatively initial stages, and several avenues of research are still open. Moreover, comparison studies are needed so as to determine the merits and demerits of the different methods in use.

## 6. Performance Evaluation

The inference accuracy can be assessed using the knowledge of a gold-standard network or the true network. In order to benchmark the algorithms, the correctly identified edges or true positives (TPs) need to be calculated. In addition, the number of false positives (FPs), or the edges incorrectly indicated to be present, and false negatives (FNs) which is the missed detection should also be counted [10]. With these values in hand, true positive rate or recall; that is, TPR = TP/(TP + FN), false positive rate; that is, FPR = FP/(FP + TN), and positive predictive value; that is, PPV = TP/(TP + FP), also called the precision, can be calculated. These quantities enable us to view the performance graphically by the area under the ROC curve which plots FPR versus the TPR. These criteria are most widely used as the fidelity criterion for gene network inference algorithms.

While it is possible to identify the gene regulatory relationships experimentally, it would not only be technically prohibitive but also proved to be very costly. For this reason, several *in silico* and *in vivo* networks have been generated to assist in benchmarking the network inference algorithms. Foremost among these are the DREAM (dialogue on reverse engineering assessment and methods) [44] and IRMA (*in vivo* reverse engineering and modeling assessment) [45] datasets. Reference [10] provides a unified survey of some of the important algorithms in gene network inference algorithms using these datasets.

## 7. Discussions and Conclusions

This paper reviews the main statistical methods used for inference of gene and protein-protein networks. PPI network inference can be carried out in a wide variety of ways by exploiting phylogenetics information and sequencing data. This paper focused only on those inference methods that employ PPI data.

For the inference of gene regulatory networks, the problem can be simply stated as follows: given the gene expression data, find the interactions between the genes. Three major classes of statistical methods were reviewed in this paper: probabilistic graphical models, state-space models, and information theoretic methods. For all these methods, modeling as well as inferencing techniques was discussed. It is observed that much progress has been made in the field of GRN inference. However, almost all of the proposed network inference methods in the literature work with only the popular gene expression data sets. An interesting part of future work could be integrating different data sets and biological knowledge available to come up with better and more robust algorithms.

Comparing the three broad classes of statistical methods reviewed in the paper, it is found that the information theoretic methods have advantages over the other methods in terms of minimal modeling assumptions and, therefore, are capable of modeling more general networks. Graphical modeling techniques assume the network to be acyclic in case of Bayesian network modeling and provide an undirected graph when using Markov networks. The state-space nonlinear models work with nonlinear functions which may not be the true representative of the underlying network, thereby resulting in less robust algorithms.

In case of PPI network prediction, the most popular statistical method is clustering. In addition, probabilistic graphical modeling techniques are also used. However, several important avenues of research are still open. Since the Markov networks and Bayesian networks are able to model PPI networks efficiently, other probabilistic graphical techniques such as factor graphs could potentially be used for solving this inference problem. Clustering methods are more suited to the PPI network inference problem as the main emphasis is on the identification of protein complexes. It is found that certain important and popular modeling techniques may fail to model PPI networks [46]. Also, clustering methods based on mutual information could be used [47].

Several statistical methods have been proposed to infer an integrated network of transcription regulation and protein-protein interaction. A state-space model for integrated network inference involves parameter estimation which indicates the strength of the inhibitory and excitatory regulations. As the cellular networks are known to be sparse, employing sparsity-constrained least squares for parameter estimation as proposed in [25] is expected to result in more robust inference algorithms.

Recent years have shown tremendous and rapid progress in the field of cellular network modeling. With the amount and types of data sets increasing, algorithms combining multiple datasets are necessary for future.
